# How to individualize renoprotective therapy in obese patients with chronic kidney disease: a commentary by the Diabesity Working Group of the ERA

**DOI:** 10.1093/ndt/gfaf069

**Published:** 2025-05-13

**Authors:** Enrique Morales, William P Martin, Sebastjan Bevc, Trond G Jenssen, Marius Miglinas, Matias Trillini

**Affiliations:** Department of Nephrology, Hospital 12 de Octubre, Madrid, Spain; Research Institute of University Hospital 12 de Octubre (imas12), Department of Medicine, Complutense University of Madrid, Madrid, Spain; Division of Nephrology, Department of Medicine, Washington University in St. Louis, St. Louis, MO, USA; Department of Nephrology, Clinic for Internal Medicine, University Medical Centre Maribor, Maribor, Slovenia; Medical Faculty, University of Maribor, Maribor, Slovenia; Department of Transplantation Medicine, Section of Nephrology, Oslo University Hospital Rikshospitalet, Oslo, Norway; Institute of Clinical Medicine, University of Oslo, Oslo, Norway; Center of Nephrology, Santaros Klinikos, Clinic of Gastroenterology, Nephrourology and Surgery, Institute of Clinical Medicine, Faculty of Medicine, Vilnius University, Vilnius, Lithuania; Istituto di Ricerche Farmacologiche Mario Negri IRCCS, Bergamo, Italy

**Keywords:** CKD, incretin, obesity, renoprotective, type 2 diabetes

## Abstract

The interrelated pandemics of obesity and type 2 diabetes mellitus (T2DM) are fuelling an increase in the prevalence of chronic kidney disease (CKD), which amplifies the risk of cardiovascular events and may progress to end-stage kidney disease (ESKD). Treatment options for such patients have rapidly expanded over the past decade and continue to evolve. Herein, we primarily focus on glucagon-like peptide-1 receptor agonists (GLP-1RAs) and their role in the management of CKD in the setting of overweight/obesity and T2DM. Recommendations from the recent Kidney Disease: Improving Global Outcomes CKD guidelines are summarized and new evidence arising since publication of these guidelines is highlighted. We review clinical studies supporting the role of GLP-1RAs in patients with diabesity and CKD, including the FLOW trial, as well as exploring potential mechanisms of their nephroprotective effects. Their role in the management of patients with ESKD on maintenance dialysis and after kidney transplantation, while less evidence-based, is also discussed. The potential for other gut hormone–based therapies, including GLP-1/glucose-dependent insulinotropic polypeptide dual agonists (tirzepatide), triple agonists (incorporating glucagon agonism) and amylin analogues to improve cardiovascular and kidney outcomes in patients with CKD, is explored. We highlight the role of novel therapies distinct from the gut–kidney axis, including non-steroidal mineralocorticoid receptor antagonists (nsMRAs). We outline the potential for multitarget therapy incorporating renin–angiotensin–aldosterone system inhibitors, sodium–glucose co-transporter-2 inhibitors, incretin-based treatments and nsMRAs to improve cardiovascular and kidney outcomes in patients with overweight/obesity and T2DM. Current unknowns in the timing and sequence of multitarget therapy in patients with CKD are emphasized. Priority research questions for the future are highlighted throughout the review.

## INTRODUCTION

The combination of obesity with or without type 2 diabetes mellitus (T2DM) and chronic kidney disease (CKD) is a global medical problem in which nephrologists should play a leading role and provide strong scientific evidence for a personalized therapeutic strategy [1]. The combined prevalence of overweight [body mass index (BMI) ≥25–30 kg/m^2^) and obesity (BMI ≥30 kg/m^2^) continues to increase. In 2020, 42% of the global adult population was predicted to be affected by overweight or obesity, a figure that is projected to increase to 54% by 2035 [2]. Overweight and obesity are disproportionately higher in patients with CKD; for instance, the combined prevalence of overweight and obesity exceeded 70% in 2018–2019 in a report from one tertiary nephrology centre in western Europe [3]. Moreover, there are numerous complications associated with obesity, depending on the stage of CKD (e.g. progression of CKD, difficulty in accessing kidney transplantation, increased cardiovascular risk, increased hospitalizations, increased healthcare costs and a reduction in quality of life and life expectancy) [4].

Recently, a commission of *The Lancet Diabetes & Endocrinology* published criteria to redefine obesity, specifically distinguishing between preclinical and clinical obesity [5]. Within this framework, clinical obesity is considered a chronic, systemic disease state caused by excess adiposity and characterized by organ dysfunction or limitation in daily activities. In contrast, preclinical obesity is a state of excess adiposity conferring increased future health risk but without current organ dysfunction or limitation in daily activities. The limitations of overreliance on BMI to define obesity are stressed by the commission, who encourage direct measurement of body fat or assessment of other anthropometric measures such as waist circumference, waist:hip ratio or waist:height ratio alongside BMI to define obesity status. It is hoped that this updated diagnostic framework will improve delivery of personalized care for individuals living with obesity.

The American Heart Association (AHA) has recently introduced a new concept combining several entities (metabolic syndrome, cardiovascular disease and CKD), known as the cardiovascular–kidney–metabolic (CKM) syndrome [6]. This new condition requires the establishment of multidisciplinary approaches and therapeutic strategies to reduce morbidity and mortality in this group of patients. Successful implementation of nephroprotective and cardioprotective therapies has the potential to improve overall health outcomes and reduce health inequalities [7]. In recent years, the results of several clinical trials have led to a new therapeutic approach to cardiovascular and renal events in obese patients with or without T2DM and CKD. Alongside renin–angiotensin–aldosterone system inhibitors (RAASis), sodium–glucose co-transporter-2 inhibitors (SGLT2is), glucagon-like peptide-1 receptor agonists (GLP-1RAs) and non-steroidal mineralocorticoid receptor antagonists (nsMRAs) have become the cornerstones of treatment for this group of patients [8].

For example, an actuarial analysis of the effects of combining RAASis, SGLT2is, GLP-1RAs and nsMRAs in patients with T2DM and albuminuria estimated a 35% reduction in major adverse cardiovascular events (MACE) relative to RAASi therapy and traditional risk factor control alone {hazard ratio [HR] 0.65 [95% confidence interval (CI) 0.55–0.76]} [9]. This translated to a 3.2-year (95% CI 2.1–4.3) increase in MACE-free survival for a 50-year-old patient. Estimates for increases in survival without other clinically relevant events were similar: hospitalized heart failure (3.2 years), CKD progression (5.5 years) and cardiovascular death (2.2 years). Similarly, the estimated lifetime benefit of combined RAASi and SGLT2i therapy in patients with albuminuric CKD without diabetes has been quantified [10]. Survival without doubling of serum creatinine, kidney failure or death for a 50-year-old patient improved from 9.6 years with no treatment to 17 years with combined treatment. Therefore, the next challenge for nephrologists is to integrate these pharmacological agents into the management strategies of obesity/overweight in people at risk of CKD or with CKD, despite the presence of T2DM, in order to achieve the endpoint of improving outcomes of CKM events.

The first section of this commentary outlines the role of GLP-1RAs in patients with CKD, end-stage kidney disease (ESKD) and kidney transplant recipients. Compared with the general CKD population, the latter two areas are not well evidence-based. We briefly discuss Kidney Disease: Improving Global Outcomes (KDIGO) diabetic kidney disease (DKD)/CKD guideline recommendations on GLP-1RAs, which are now largely outdated, before exploring putative mechanisms of the nephroprotective effects of GLP-1RAs. Subsequently, the evidence for other gut hormone–derived therapies in patients with CKD is discussed. Finally, we highlight unanswered questions and priorities for future research, including multitarget therapy to improve cardiovascular and kidney outcomes in patients with CKD.

## ROLE OF GLP-1RAS IN SPECIFIC POPULATIONS WITH KIDNEY DISEASE

### CKD in the setting of overweight, obesity or T2DM

The nephroprotective effects of GLP-1RAs in CKD were initially observed in cardiovascular outcomes trials (CVOTs) of GLP-1RAs in patients with T2DM and high cardiovascular risk (Table 1) [11–17]. The signal for nephroprotection, particularly with respect to reducing new-onset macroalbuminuria, accumulated from these studies, resulted in the design of the FLOW trial (NCT03819153), the first clinical trial of a GLP-1RA (semaglutide 1 mg weekly) with a primary kidney endpoint [18]. Additionally, semaglutide 2.4 mg weekly was found to be nephroprotective in patients with overweight/obesity and at high cardiovascular risk, but without T2DM, in the SELECT trial (NCT03574597) [19]. Similarly, in the SMART trial (NCT04889183), semaglutide 2.4 mg weekly lowered albuminuria by ≈50% from baseline over a 24-week treatment period in patients with overweight/obesity and CKD, but without T2DM [20]. The impact of GLP-1RAs compared with placebo on kidney disease endpoints including albuminuria, eGFR decline, incidence of ESKD and composite kidney/cardiovascular endpoints in these CVOTs and in primary kidney outcome studies is summarized in Table 1.

**Table 1:** Effect of GLP-1RAs compared with placebo on kidney endpoints in CVOTs (patients with overweight/obesity and/or T2DM and high cardiovascular risk) and in primary kidney outcome studies (patients with overweight/obesity and/or T2DM and CKD).

			Kidney outcomes			
Trial CVOTs	Agent (all versus placebo)	Year	New-onset macroalbuminuria	eGFR decline	ESKD	Composite endpoints
T2DM and high cardiovascular risk						
ELIXA [11]	Lixisenatide	2015	0.84 (0.68–1.02)^a^	1.16 (0.74–1.83) (DSCr)	NR	NR
SUSTAIN-6 [12]	Semaglutide 0.5 or 1 mg once weekly	2016	**0.54 (0.37–0.77)**	1.28 (0.64–2.58) (DSCr and eGFR <45)	0.91 (0.40–2.07) (KRT)	**0.64 (0.46–0.88)** (macroalbuminuria, DSCr and eGFR <45, KRT or kidney death)
EXSCEL [13]	Exenatide extended-release once weekly	2017	0.87 (0.70–1.07)	NR	NR	0.88 (0.76–1.01) (macroalbuminuria, ≥40% eGFR decline, KRT or kidney death)
LEADER [14]	Liraglutide	2016 (original study), 2017 (report on renal outcomes)	**0.74 (0.60–0.91)**	0.89 (0.67–1.19) (DSCr and eGFR <45)	0.87 (0.61–1.24) (KRT)	**0.78 (0.67–0.92)** (macroalbuminuria, DSCr and eGFR <45, KRT or kidney death)
HARMONY [15]	Albiglutide	2018	NR	−1.11 (−1.84 to −0.39) lower with GLP-1RA at 8 months −0.43 (−1.26–0.41) lower with GLP-1RA at 16 months (eGFR difference)	NR	NR
REWIND [16]	Dulaglutide	2019	**0.77 (0.68–0.87)**	0.89 (0.78–1.01) (≥30% eGFR decline)	0.75 (0.39–1.44) (KRT)	**0.85 (0.77–0.93)** (macroalbuminuria, ≥30% eGFR decline or KRT)
AMPLITUDE-O [17]	Efpeglenatide	2021	**0.68 (0.58–0.80)**	**−2.37 GLP-1RA versus −3.26 placebo** Difference 0.89 (0.27–1.51) (eGFR at 24 months)	NR	**0.68 (0.57–0.79)** (macroalbuminuria + increase in UACR ≥30% from baseline, ≥40% eGFR decline, KRT or eGFR <15)
Overweight/obesity and high cardiovascular risk without T2DM						
SELECT [19]	Semaglutide 2.4 mg once weekly	2024	0.80 (0.64–1.00)	0.57 (0.27–1.14) (≥50% eGFR decline) **−0.78 GLP-1RA versus −1.17 placebo (*P* < .001)** (annual eGFR change − total eGFR slope)	1.24 (0.33–5.02) (eGFR <15) 0.66 (0.17–2.32) (KRT)	**0.78 (0.63–0.96)** (macroalbuminuria, ≥50% eGFR decline, eGFR <15, KRT or kidney death) **0.82 (0.69–0.97)** (≥50% eGFR decline, eGFR <15, KRT, cardiovascular death or kidney death)
Primary kidney outcome studies			Change in UACR	eGFR decline	ESKD	Composite endpoints
T2DM and CKD						
FLOW [18]	Semaglutide 1 mg once weekly	2024	ETR **0.68 (95% CI 0.62–0.75)** in favour of semaglutide for UACR at week 104 relative to baseline Ratio of UACR at week 104 to baseline was **32% (95% CI 25–38)** lower in the semaglutide group than in the placebo group	**0.73 (0.59–0.89)** (≥50% eGFR decline) **−2.19 GLP-1RA versus −3.36 placebo (*P* < .001)** (annual eGFR change − total eGFR slope)	0.80 (0.61–1.06) (eGFR <15) 0.84 (0.63–1.12) (KRT)	**0.76 (0.66–0.88)** (≥50% eGFR decline, eGFR <15, KRT, cardiovascular death, or kidney death) **0.79 (0.66–0.94)** (≥50% eGFR decline, eGFR <15, KRT, or kidney death)
Overweight/obesity and CKD without T2DM						
SMART [20]	Semaglutide 2.4 mg once weekly	2025	Placebo-corrected geometric mean change in UACR at week 24 of **−52.1% (95% CI −65.2 to −34.1)** in favour of semaglutide	No difference between semaglutide and placebo in eGFR (assessed by creatinine and cystatin C) and subgroup with iohexol mGFR at week 24	NR	NR

DSCr: doubling of serum creatinine; ETR: estimated treatment ratio; KRT: kidney replacement therapy (chronic dialysis or kidney transplantation); mGFR: measured glomerular filtration rate; NR: not reported.

Statistically significant results in bold.

Data are presented as HR (95% CI) unless stated otherwise.

Definitions are provided in parentheses beneath the data within table cells for outcomes with heterogeneity between studies (eGFR decline, ESKD and composite endpoints).

^a^This result reached statistical significance when adjusted for baseline HbA1c [HR 0.81 (95% CI 0.66–0.99)].

It is notable from Table 1 that GLP-1RAs primarily reduced incident macroalbuminuria in secondary outcome and post hoc analyses of CVOTs, while the effect on the estimated glomerular filtration rate (eGFR) slope was less consistent. Nevertheless, a trend towards slower eGFR decline in patients with T2DM was observed with dulaglutide in the REWIND trial (NCT01394952) and with efpeglenatide in the AMPLITUDE-O trial (NCT03496298) [16, 17]. Similarly, a slower rate of annual eGFR decline in patients with overweight/obesity was observed with semaglutide in the SELECT trial [19]. GLP-1RAs also reduced the incidence of composite kidney endpoints in these CVOTs, in large part driven by the reduced incidence of macroalbuminuria. The CVOTs of GLP-1RAs outlined in Table 1 were conducted in populations at high cardiovascular risk but not at high risk of developing clinically relevant kidney disease endpoints. These study populations primarily consisted of patients without CKD or with early stage CKD. As such, power to detect a meaningful impact of GLP-1RAs on the rate of eGFR decline, CKD progression and incidence of ESKD was limited in these studies as the event rates were low.

The FLOW trial randomized patients with T2DM at high risk of CKD progression to semaglutide 1 mg weekly or placebo to determine the impact of semaglutide on clinically relevant kidney disease endpoints [18]. Patients at high risk of CKD progression were defined as having an eGFR of 50–75 ml/min/BSA and a urine albumin:creatinine ratio (UACR) of 300–5000 mg/g or an eGFR of 25–50 ml/min/BSA and a UACR of 100–5000 mg/g. The proportion of patients with an eGFR >60 ml/min/BSA was capped at 20% of the study population. The mean eGFR was 47 ± 15 ml/min/BSA and the median UACR was 568 mg/g at enrolment. More than 95% and 15% of the study population were on a RAASi and SGLT2i at enrolment, respectively. No data on nsMRAs were provided; it is assumed that no patients were taking finerenone at enrolment. No BMI eligibility criterion was imposed, but the mean BMI of the study population was in the World Health Organization class 1 obesity category at 32 ± 6 kg/m^2^. The incidence of the primary kidney outcome (a composite of ≥50% eGFR decline, ESKD or death from kidney or cardiovascular causes) was reduced by 24% for semaglutide relative to placebo. After excluding cardiovascular death from the composite endpoint, the effect estimate was similar, with a 21% reduction observed in the semaglutide arm. Semaglutide also lowered the annual rate of eGFR decline (total slope) by >1 ml/min/BSA/year compared with placebo.

With the promising results of primary kidney outcome studies of GLP-1RAs (the FLOW and SMART trials) and continued secondary outcome analyses of GLP-1RA CVOTs, the role of GLP-1RAs in improving cardiovascular and kidney outcomes in patients with overweight/obesity or T2DM and CKD appears to be expanding rapidly. However, many priority research questions remain. The impact of GLP-1RAs on kidney endpoints in type 1 DM, non-albuminuric type 2 DKD, non-diabetic CKD and in CKD arising in the context of normal body weight remains to be fully defined. The results of the SMART trial are promising with respect to the application of GLP-1RAs to non-diabetic CKD [20]. However, this was a small trial (101 patients randomized to semaglutide or placebo) with a short duration of follow-up (24 weeks) that demonstrated a positive impact of semaglutide on albuminuria. Larger and longer-term studies are needed to test the impact of GLP-1RAs on the GFR slope and other more clinically relevant kidney endpoints in non-diabetic CKD. The impact of GLP-1RAs in underrepresented populations at high risk of CKD progression, such as Black patients, needs to be studied further. The timing and sequence of how to combine GLP-1RAs with other goal-directed therapies for CKD also needs further study, as does how they might synergize with metabolic/obesity surgery to provide durable improvements in cardiovascular and kidney outcomes.

### ESKD

Patients with advanced CKD (eGFR <30 ml/min/BSA) and ESKD have largely been excluded from CVOTs of GLP-1RAs. For example, participants with an eGFR <30 ml/min/BSA constituted <0.5% of the SELECT trial population [21]. In the first clinical trial with a primary kidney outcome (FLOW), participants could be enrolled down to an eGFR of 25 ml/min/BSA and participants with an eGFR <30 ml/min/BSA constituted 11% of the study population [18]. As such, the impact of GLP-1RAs on cardiovascular and kidney outcomes is limited in the setting of advanced CKD and ESKD, and this should be a priority for future studies.

Separate from their end-organ benefits on the heart and kidney, GLP-1RAs may also play an important role in weight management in patients with ESKD. Although higher BMI is associated with lower mortality in people with ESKD on maintenance dialysis [22], obesity is a major barrier to kidney transplantation in people with ESKD. Most kidney transplant centres consider BMIs >40 kg/m^2^ and >35 kg/m^2^ absolute and relative contraindications, respectively, to waitlisting for kidney transplantation [23]. GLP-1RAs may improve rates of waitlisting for kidney transplantation by effecting intentional weight loss in patients with ESKD.

Existing data on the use of GLP-1RAs in patients with ESKD are derived from small randomized controlled trials (RCTs) and retrospective cohort studies, most of which used liraglutide [24]. The largest randomized study treated 20 people with type 2 diabetes and ESKD and 20 control patients with type 2 diabetes not complicated by kidney disease with liraglutide or placebo [25]. Over a 12-week period, liraglutide improved glycaemic control and reduced body weight in people with and without ESKD. The magnitude of weight loss was greater in patients without kidney disease than in those with ESKD, with body weight decreasing by 2.4 ± 0.8 kg in those with ESKD (*P* = .22). Plasma trough liraglutide concentrations were higher and gastrointestinal (GI) side effects occurred more frequently in the ESKD group.

A retrospective observational cohort study between 2018 and 2023 evaluated the use of oral (18%) and injectable (82%) semaglutide in patients with CKD stage 4 (*n* = 63), CKD stage 5 (*n* = 1) and ESKD on maintenance dialysis (n = 12; *n* = 11 HD, *n* = 1 peritoneal dialysis) [26]. The median duration of semaglutide therapy was 17.4 months (interquartile range 0.4–48.8). Body weight decreased by an average of 5.1 kg, corresponding to a 4.6% loss of baseline body weight. Of the 96% of the study population who had T2DM, the average haemoglobin A1c (HbA1c) reduction was 0.9%. Hypoglycaemia occurred in 20 (26%) patients. Eight patients discontinued insulin after starting semaglutide. Of the 27 patients who discontinued semaglutide, 10 (37%) discontinued due to GI side effects.

GI side effects, including constipation, are commonly encountered with GLP-1RAs, particularly during the dose titration phase [27]. From the limited available evidence, these side effects may be more common in patients with ESKD and the tolerability of GLP-1RAs will be an important determinant of their clinical utility in this population [24]. Slower dose titration may be preferable. GLP-1RA-induced constipation may be particularly problematic in patients on peritoneal dialysis, and a proactive bowel regimen should be instituted to prevent catheter-related complications [28]. The risk of hypoglycaemia is likely to be increased in patients with ESKD; other glucose-lowering therapies, particularly insulin, should be down-titrated or discontinued as GLP-1RAs are started [26]. Frequent snacking to counter hypoglycaemia may counter weight loss.

### Kidney transplant

Unlike a patient with CKD, a kidney transplant patient has a single, denervated kidney, in most cases being exposed to nephrotoxic drugs, e.g. calcineurin inhibitors. Accordingly, data from CKD studies cannot be extrapolated to renal transplant recipients.

It is not known if hyperfiltration occurs at the single-nephron level in a healthy transplanted kidney with presumably intact autoregulation. The current belief is that autoregulation on the glomerular level is independent of external kidney innervation. In fact, in a small study of kidney transplant recipients treated with the SGLT2 inhibitor dapagliflozin, an early decrease in GFR, as measured by iohexol clearance, was detected in these patients [29]. This would indicate that haemodynamic responses are intact in a denervated kidney.

Nephroprotection with GLP1-RAs could take place both through reduced hydrogen–sodium antiporter activity, which increases natriuresis [30], and also through a reduction in total body mass, ectopic fat and blood pressure (BP), which might alleviate filtration pressure in glomerular filtration tufts. However, to date, we have neither experimental nor clinical data to prove that this is the case. No RCTs have been performed with GLP1-RAs in kidney transplant recipients, although studies are on their way. However, we do have available small observational studies of limited observation time (<12 months), as reviewed by Valencia-Morales *et al.* in 2023 [31]. Twelve observational studies in solid organ transplant patients (of which eight studies were performed in kidney transplant recipients) all reported that the use of GLP1-RAs was primarily associated with body weight loss (2–5 kg) and a corresponding decrease in HbA1c. On the other hand, marginal and inconsistent effects on eGFR (not measured) were reported, but trough levels of tacrolimus were in general not affected. Side effects were identical and just as frequent as those reported in non-transplanted patients [31]. Two small observational studies [32, 33] found a reduction in UACR with GLP1-RAs in kidney transplant recipients, but this has not been measured or confirmed in other studies.

In a recent observational study on 318 solid organ transplant recipients (of which 272 patients received a kidney transplant), the use of GLP1-RAs was associated with a reduced occurrence of MACE and all-cause mortality [34]. However, this finding has to be verified by RCTs.

## LESSONS FROM 2022 (DKD) AND 2024 (CKD) KDIGO GUIDELINES: WHAT ROLE DO GLP-1RAS PLAY IN THE KDIGO CKD GUIDELINES?

Published in 2022, the KDIGO Clinical Practice Guidelines for Diabetes Management in Chronic Kidney Disease provided recommendations for non-pharmacological and pharmacological interventions in patients with diabetes and CKD [35]. Following lifestyle measures, and with some specific considerations regarding remaining/residual kidney function, the KDIGO guidelines emphasized that the first-line pharmacological treatments for patients with kidney disease associated with T2DM should include metformin, SGLT2is, RAASis, ACEis or ARBs and statins.

At the time of guideline publication in the pre-FLOW trial era [18], GLP-1RAs remained a second-line option that may be a preferred treatment for patients with T2DM and CKD at high risk for cardiovascular events, with residual albuminuria or in need of intensification of glycaemic control or assistance with intentional weight loss efforts. The guidelines appropriately delineated the common side effects of GLP-1RAs, which are predominantly GI and largely dose dependent. To minimize GI side effects, it is advised to slowly up-titrate GLP-1RAs. Injection site reactions are also recognized with subcutaneous formulations of these drugs. The risk of hypoglycaemia is low, but attention to concomitant glucose-lowering therapies with a higher propensity for hypoglycaemia (insulin, sulphonylureas) is critical when starting GLP-1RAs, particularly in patients with CKD. Guidelines do not support combining dipeptidyl peptidase-4 inhibitors with GLP-1RAs due to a lack of additional clinical benefit.

The KDIGO 2024 Clinical Practice Guideline for the Evaluation and Management of Chronic Kidney Disease was published in April 2024 [36]. At the time of publication, the FLOW trial had not yet been published [18]. Therefore, available information regarding the use of GLP-1RAs in patients with DKD was not substantially different from when the 2022 diabetes and CKD update was published [35].

In view of the FLOW trial, GLP-1RAs will be front and centre alongside RAASis, SGLT2is and nsMRAs as the four pillars for cardiovascular and kidney protection in future CKD guidelines.

## GLP-1RAs: MECHANISMS OF ACTION

GLP-1RAs improve intermediary determinants of both adverse cardiovascular and kidney outcomes in people with overweight/obesity and/or T2DM, including body weight, BP, glycaemia and circulating lipids. Several CVOTs, outlined in Table 1, have demonstrated their benefit on atherosclerotic cardiovascular disease in this population. The FLOW RCT also highlighted the cardiovascular protection afforded by semaglutide in people with T2DM and more advanced CKD [18]. The vasculoprotective and cardioprotective effects of GLP-1RAs should contribute to reductions in atherosclerotic renal vascular disease and cardiorenal syndrome in people with obesity and/or T2DM. Preclinical and clinical data suggest that GLP-1RAs improve endothelial function [37]. GLP-1RAs lower albuminuria (observed in GLP-1RA CVOTs and in the FLOW RCT), which minimizes tubular damage and CKD progression.

**Figure 1:**
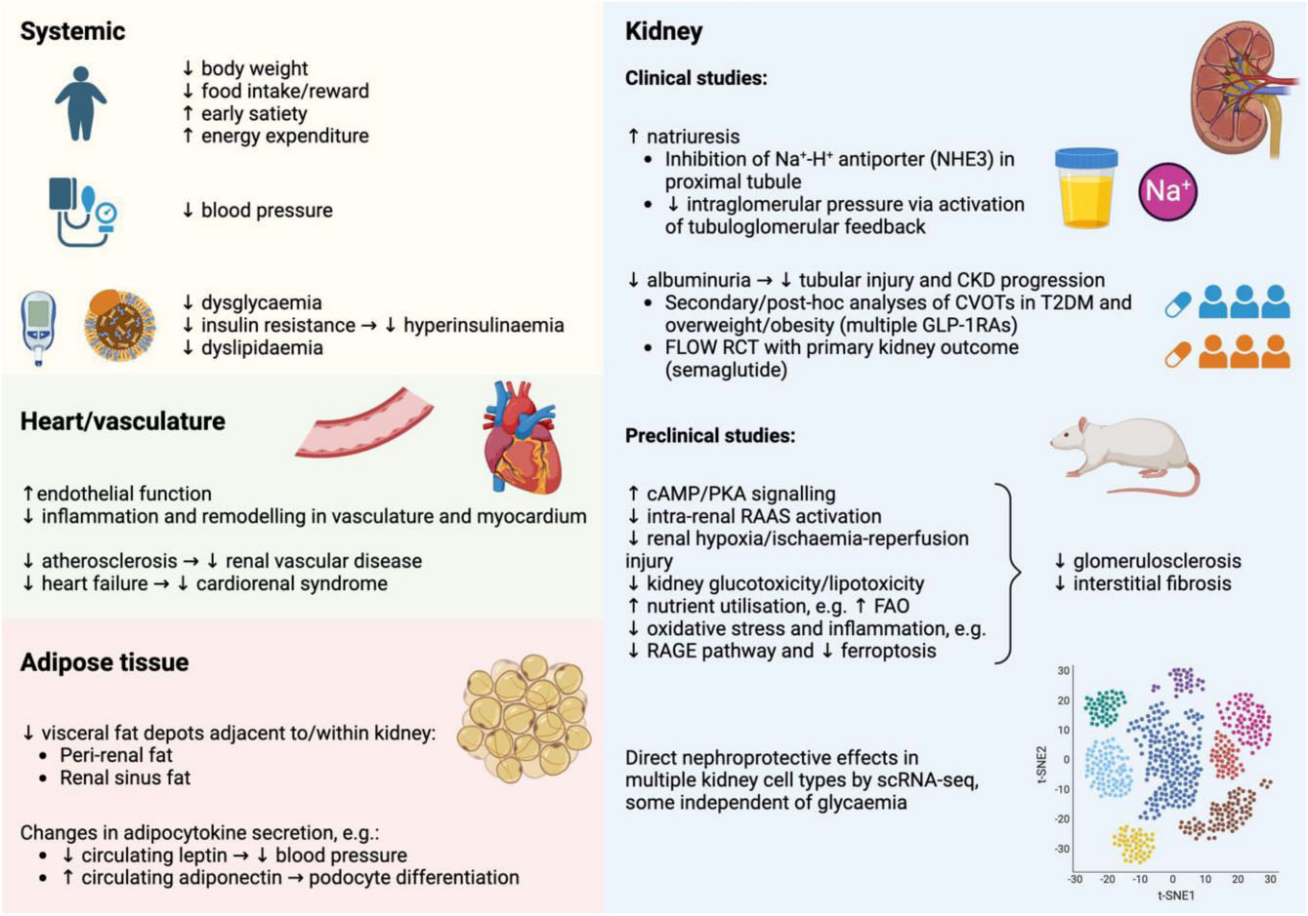
Putative mechanisms of GLP-1RA-induced nephroprotection. cAMP: cyclic adenosine monophosphate; FAO: fatty acid oxidation; GLP-1RA, glucagon-like peptide-1 receptor agonist; PKA: protein kinase A; RAGE: receptor for advanced glycation end-products; scRNA-seq: single-cell RNA sequencing.

Additional mechanisms have been implicated in the nephroprotective effects of GLP-1RAs (Fig. 1). Some of these are extrapolated from other relevant fields of study, such as metabolic/obesity surgery wherein increased endogenous GLP-1 signalling observed postoperatively is central to nephroprotection [38]. It is also noteworthy that GLP-1RAs appear to directly counter many of the pathophysiological mechanisms of CKD arising in the context of obesity and T2DM. Detailed discussion of molecular mechanisms and cellular signalling activated by GLP-1RAs is beyond the scope of the current commentary; we refer the interested reader to a relevant review [39].

The accumulation of perirenal and renal sinus fat that occurs in patients with obesity and T2DM has been associated with hypertension and CKD [40]. For example, renal sinus fat area was quantified by computed tomography in 2923 adults enrolled in the Framingham Heart Study and the prevalence of ‘fatty kidney’ (renal sinus fat area >90th percentile) was 30% [41]. Individuals with ‘fatty kidney’ were at increased risk of hypertension and CKD despite adjustment for BMI and abdominal visceral adipose tissue [41]. Purported mechanisms of how perirenal fat may contribute to adverse CKD outcomes include mechanical compression of the kidney, changes in glomerular haemodynamics including increased afferent arteriolar resistance and increased renal vascular resistance, increased renal tubular reabsorption of sodium, paracrine secretion of pro-inflammatory cytokines and vasoconstrictive factors and lipotoxicity mediated by lipolysis and local fatty acids release [40, 42]. Reductions in perirenal and renal sinus fat accompanying weight loss may reduce BP and kidney injury. Greater reductions in renal sinus fat after weight loss are associated with improved BP control in people with obesity [43]. Changes in adipocytokine secretion accompanying weight loss may also exert nephroprotective actions. Obesity is characterized by low circulating adiponectin levels that impair insulin signalling and secretion [44]. Higher plasma adiponectin levels correspond to greater rates of T2DM remission after weight loss in people with obesity [45]. Additionally, murine models have demonstrated that adiponectin promotes podocyte differentiation, providing a direct link between adipose tissue health and glomerular permeability [46].

It has also been hypothesized that GLP-1RAs may improve glomerular haemodynamics by inhibiting the sodium–hydrogen antiporter 3 (NHE3) in the proximal tubule, causing natriuresis and lowering intraglomerular pressure via tubuloglomerular feedback. Increased urinary sodium excretion has been observed after infusion of GLP-1 and GLP-1RAs in people with obesity [47–49]. An 8-week treatment with the GLP-1RA lixisenatide in patients with T2DM increased urinary sodium excretion and the amount of NHE3 in urinary extracellular vesicles, suggesting decreased NHE3 activity in the proximal tubule [48]. It should be noted, however, that there was no difference in the acute 12-week eGFR slope between the semaglutide and placebo arms in the FLOW RCT [18], suggesting that changes in glomerular haemodynamics may be less pronounced than those observed after SGLT2i initiation.

Other mechanisms of nephroprotection have largely been elucidated in preclinical models and include activation of cyclic adenosine monophosphate/protein kinase A signalling and diminished intrarenal RAAS activation [50]. Single-cell transcriptomic analyses in rodent models of DKD and non-diabetic CKD (subtotal nephrectomy) have demonstrated glycaemia-independent benefits of GLP-1RAs on multiple kidney cell types including glomerular endothelial cells, podocytes, proximal tubular cells and macrophages [51]. Pathway-level changes were notable for downregulation of the receptor for advanced glycation end-products pathway and a shift towards protective fatty oxidation in proximal tubular and endothelial cells [51]. The latter finding suggests that GLP-1RAs may counter DKD progression by improving kidney nutrient utilization and mitochondrial function. Similarly, peroxisome proliferator-activated receptor α–stimulated proximal tubular fatty acid oxidation has been identified as a key mechanism underpinning the nephroprotective effects of intentional weight loss plus multimodal pharmacotherapy (including GLP-1RAs) in animal models of DKD [52, 53]. Collectively, the data suggest that GLP-1RAs may have a beneficial impact on bioenergetics in the proximal tubule.

## OTHER GUT HORMONE–DERIVED THERAPIES

### Tirzepatide

Tirzepatide is a dual GLP-1 and glucose-dependent insulinotropic polypeptide (GIP) receptor agonist. It is a 39 amino acid chain connected to a C20 fatty acid chain in order to prolong its half-life [54]. The polypeptide is primarily a GIP receptor ligand but modified also to stimulate the GLP-1 receptor [55]. An additive effect with GIP receptor activation is to increase insulin sensitivity in white fat tissue, which in turn lowers plasma fatty acids as well as triglycerides. This improves insulin sensitivity in addition to the effects of GLP-1R activation [56]. This in turn increases the loss of body weight and glucose lowering. However, GIP receptors have not been located in the kidneys, so any extra effect of tirzepatide on the kidneys must be due to desirable extrarenal effects, including a reduction in body weight, reduced glycaemia in diabetes and prediabetes, lowered BP and reversal of insulin resistance.

No primary studies with tirzepatide in CKD are available. However, in the SURPASS program, which addresses the glucose- and weight-lowering properties of tirzepatide in patients with T2DM, the UACR was found to be lowered compared with comparators such as placebo or insulin. In fact, a post hoc analysis of the phase 3 SURPASS-4 study (NCT03730662) was clearly associated with beneficial kidney outcomes [57]. In this study, 2002 eligible patients with T2DM that was inadequately controlled with any combination of sulfonylurea, metformin or SGLT2 inhibitor were randomized in a 1:1:1:3 ratio to either treatment with three different weekly doses of tirzepatide (5 mg, 10 mg or 15 mg) or daily insulin glargine. At baseline, the median eGFR was 81 ml/min/BSA (18% had an eGFR <60 ml/min/BSA), median UACR was 15 mg/g and average BMI was 32.6 kg/m^2^. Furthermore, baseline HbA1c was 8.5% and 25% of the patients used an SGLT2 inhibitor. In the combined tirzepatide groups (all doses taken together), the UACR did not increase over the median treatment duration of 85 weeks while it increased by 37% from baseline over the same period in those treated with insulin glargine. The eGFR slope was −1.4 ml/min/BSA/year in the combined tirzepatide groups compared with −3.6 ml/min/BSA/year in the insulin glargine group. Furthermore, patients treated with tirzepatide were less likely to develop the composite kidney endpoint (eGFR decline of ≥40%, new-onset macroalbuminuria, ESKD or renal death), an effect that was mainly driven by the effect on macroalbuminuria.

### Glucagon and amylin

There are other nutrient-stimulated hormone-based anti-obesity medications in development. These new pharmaceuticals are based on stimulating (or inhibiting) the action of one or more gut hormones, including GLP-1, GIP, glucagon and amylin.

Glucagon promotes lipolysis in the liver and may increase energy expenditure. There is emerging evidence of the efficacy on weight reduction of dual GLP-1R/glucagon receptor (GCGR) agonists (cotadutide, mazdutide, survodutide) [58–60] and triple GLP-1R/GIP receptor/GCGR agonists (retatrutide) in overweight/obese individuals with or without diabetes. Of note, the weight reduction with the triple agonist retatrutide has been shown to be almost as high as with surgical interventions (24.2% after 48 weeks) [61].

There is an abundance of GCGR in the kidney. The expression of these receptors in the kidney is second only to the liver. Activation of GCGR by analogues can directly and indirectly influence kidney function and lead to increased GFR, natriuresis and diuresis [62]. A combination of GCGR agonism and incretin receptor agonism therefore looks promising for a beneficial impact on kidney function, such as albuminuria reduction with cotadutide [63]. Kidney benefits of the triple agonist–based anti-obesity management strategy is an active area of investigation as well. A phase 2 clinical trial is in progress to explore the effect of retatrutide on kidney outcomes in participants with overweight/obesity and CKD with or without diabetes [64].

Amylin, which is released by the beta cells of the pancreas at the same time as insulin, improves sensitivity to leptin and has a direct activation in the brain to promote the feeling of satiety after meals. The second-generation amylin analogue cagrilintide in combination with semaglutide was well tolerated, safe and very effective (15.6% weight loss with combination compared with 5.1% and 8.1% with semaglutide and cagrilintide alone, respectively) in a phase 2 trial [65]. Another amylin analogue, petrelintide, is under investigation. It demonstrated improved GI tolerance after repeated intake in humans [66]. Amycretin, an amylin analogue and GLP-1R agonist, caused significant (13.1%) weight loss after 3 months in a recent phase 1 study [67]. However, the impact of these new compounds on kidney outcomes has not yet been reported.

## FUTURE PERSPECTIVES

### Unanswered questions with respect to GLP1-RAs and kidney disease

Recently, GLP-1RAs have emerged as key players across the CKM spectrum: overweight/obesity, T2DM and associated CKD and cardiovascular disease [8]. However, as in other areas of medicine, when new drugs appear that modify the natural course of the disease, many gaps in knowledge emerge. Examples include: do GLP-1RAs provide additional nephroprotection in patients with T2DM and CKD on RAAS blockade and SGLT2 inhibitors?; what is the optimal duration of treatment with GLP-1RAs?; is there a BMI threshold below which GLP-1RAs are ineffective?; could GLP-1RAs have a role in kidney transplantation?; what about the nephroprotective effects of GLP-1RAs in non-diabetic kidney diseases?; and what other pleiotropic effects do GLP-1RAs have?

Dialysis patients are at high cardiovascular risk and the burden of CKM syndrome is critical. There is little information in the literature on the safety and efficacy of these drugs in dialysis patients [68]. Recent observational studies suggest that in the small percentage of patients who receive them, GLP-1RAs were associated with lower all-cause mortality [69]. Future studies in this vulnerable population are needed to determine the cardiovascular benefits of this treatment.

A promising and optimistic world is opening up for the application of GLP-1RAs in renal disease as well as in other pathological conditions (metabolic dysfunction-associated steatotic liver disease, neurodegenerative disorders, obstructive sleep apnoea, polycystic ovaries, addictions etc.) [70].

### RAASis and MRAs as anti-obesity drugs

RAASis and nsMRAs are not primarily accepted and classified as anti-obesity drugs. However, both groups of drugs may indirectly address obesity through their obesity-related pathways, which affect adipose tissue function, inflammation and metabolic abnormality.

Overactivation of the RAAS in obesity increases angiotensin II and aldosterone levels, which may contribute to adipogenesis, inflammation and impaired insulin sensitivity. RAASis (ACEis and ARBs) inhibit the effects of angiotensin II and aldosterone, which may reduce adipogenesis and fat accumulation [71–73]. Angiotensin II impairs insulin sensitivity by increasing oxidative stress and inflammation in metabolic tissues. RAASis can improve insulin sensitivity and glucose uptake, which could indirectly lead to weight loss and fat reduction. Furthermore, angiotensin II may influence central pathways in the hypothalamus that regulate appetite and satiety. RAASis could modulate these pathways and potentially affect food intake.

The potential of nsMRA (finerenone) as an anti-obesity drug is not well established but may be expected through some known mechanisms. Mineralocorticoid receptors in adipose tissue are involved in processes contributing to adipogenesis, inflammation and metabolic abnormalities. Finerenone affects signalling pathways of inflammation. Furthermore, finerenone may help regulate adipose tissue function by antagonising mineralocorticoid receptors. However, there is no substantial evidence from preclinical and clinical trials to directly support the use of finerenone as an anti-obesity drug. Finerenone does not directly affect weight loss, fat reduction, appetite regulation or energy consumption but may indirectly support weight management through metabolic benefits [74].

### Multitarget nephroprotective therapy (RAASi + SGLT2i + GLP-1RA + MRA)

For more than 4 decades, strong evidence has confirmed that RAASis, including ACEis and ARBs, improve renal outcomes in patients with kidney disease due to diabetes and other causes by reducing proteinuria and progression towards ESKD [36]. Said protective kidney effects were obtained along with a significant reduction in cardiovascular risk [75]. Regrettably, a small number of human studies with contradicting results have evaluated the specific effects of RAASis in slowing the progression of CKD in patients with obesity [76, 77]. In a real-world primary care database, Cohen *et al.* [78] found no difference in renal and mortality outcomes in patients with obesity and hypertension but without diabetes treated with ACEis and ARBs when compared with other antihypertensive agents. Thus, despite the known increased RAAS activity in patients with obesity and CKD, treatment with RAASis may not confer additional nephroprotection over other antihypertensives.

In the following years, several trials tested the effects of dual RAAS blockade (combined therapy with ACEi + ARB) in patients with and without DKD, which failed to enhance CKD protection and caused increased adverse events [79, 80]. However, stagnation regarding additional CKD treatments beyond RAASis ended with the advent of SGLT2is [81]. As with ACEis and ARBs, SGLT2is improve kidney and cardiovascular outcomes in patients with CKD with and without diabetes [82]. Several studies have consistently shown that patients taking SGLT2is experience modest weight loss, which can be clinically meaningful when compared with placebo or other glucose-lowering therapies [83]. Network meta-analyses reveal an average weight reduction of ≈1.5–2 kg with SGLT2is compared with placebo, and this can be sustained for up to 4 years [83].

In recent years, GLP-1RAs have demonstrated therapeutic applications beyond traditional metabolic disorders such as obesity and T2DM. These agents lower cardiovascular death, with a favourable benefit–risk profile in patients with and without diabetes [84]. Weight loss in the FLOW trial was also 4.1 kg greater with semaglutide compared with placebo over a median 2.4-year follow-up in a population with a baseline BMI of 31 kg/m^2^ [18]. The majority of patients enrolled in phase 3 RCTs of GLP-1RAs for cardiovascular and kidney endpoints were either overweight or obese [8]. Thus semaglutide and other GLP-1RAs are promising therapies for the treatment of patients with obesity and CKD.

Given the complementary mechanisms of action regarding glycaemic control and the strong evidence supporting the cardiovascular and kidney benefits of SGLT2is and GLP-1RAs in patients with T2DM and established cardiovascular disease, it is speculated that an approach using both glucose-lowering agents may have additive cardiovascular and kidney benefits [85]. This was tested to some degree in the FLOW trial (15.6% baseline SGLT2i use) [18], and post hoc analyses in patients with and without SGLT2is have been published [86]. However, the FLOW trial was not designed to test the combination of SGLT2i and GLP-1RA therapy in CKD; prospective RCTs are needed to investigate this further.

It is important to mention that almost all patients included in SGLT2i and GLP-1RA trials were on background ACEis or ARBs that are considered the standard of care for the treatment of patients with diabetes and CKD. Regrettably, real-life studies have shown that treatments that reduce cardiovascular and kidney outcomes in patients with diabetes and CKD have historically been underused, particularly in advanced disease [87, 88]. This condition is probably increased with real-life use of SGLT2is and GLP-1RAs, treatments that retain a higher level of discontinuation compared with RAASis [89].

Although with a reduced magnitude of benefit, the nsMRA finerenone improved kidney and cardiovascular outcomes in patients with T2DM and CKD on a background of RAASi therapy [90, 91], similar to SGLT2is and GLP-1RAs. A post hoc analysis of the FIDELIO-DKD (NCT02540993) and FIGARO-DKD (NCT02545049) trials, the pooled FIDELITY analysis, showed that in >7000 patients with CKD due to T2DM, the reduction in kidney and cardiovascular events by finerenone was not affected by varying severities of obesity [92]. Finerenone is currently recommended by guidelines as an additional drug treatment with specific kidney and cardiovascular protection in patients with T2DM and CKD [35]. The current real-life use of finerenone is probably lower than other standards of care for CKD, but its use in combination with RAASi, SGLT2i and GLP-1RA deserves attention in future studies.

To conclude, a multitarget approach for the treatment of CKD in patients with obesity and with or without diabetes is of potential additive effect in terms and cardiovascular and kidney protection. Future clinical trials should test the effects of combining these therapies in patients with obesity, T2DM and CKD who have high cardiovascular and kidney risk.

## CONCLUSIONS

This is the new era of therapies aimed at minimizing the deleterious effects of the CKM syndrome. Despite the limitations, there is widespread scientific evidence of the potential of new drugs (SGLT2is, GLP-1RAs and nsMRAs) to improve overall health outcomes and ameliorate health inequalities. In the coming years, we are likely to see the advent of so-called triple agonists (GLP-1–GIP–glucagon) and additional combinatory gut hormone analogue therapies are likely to emerge, promising even greater effects on weight loss and cardiometabolic health. For this reason, the point of view of nephrologists is key to the development of new trials and the positioning of this new therapeutic armamentarium in our patients with CKD at high risk of cardiovascular disease.

## Data Availability

All data presented in this review are published and available in the referenced sources. No additional source data are required.
